# How the Topology of the Mitochondrial Inner Membrane Modulates ATP Production

**DOI:** 10.3390/cells14040257

**Published:** 2025-02-11

**Authors:** Raquel Adams, Nasrin Afzal, Mohsin Saleet Jafri, Carmen A. Mannella

**Affiliations:** 1 School of Systems Biology, George Mason University, Fairfax, VA 22030, USA; radams12@gmu.edu (R.A.); nafzal@gmu.edu (N.A.); 2 Center for Biomedical Engineering and Technology, University of Maryland School of Medicine, Baltimore, MD 20201, USA; 3 Department of Pharmacology, Physiology and Drug Development, University of Maryland School of Medicine, Baltimore, MD 20201, USA

**Keywords:** mitochondria, cristae, electron tomography, metabolic modeling, membrane topology, metabolite diffusion, ATP synthesis

## Abstract

Cells in heart muscle need to generate ATP at or near peak capacity to meet their energy demands. Over 90% of this ATP comes from mitochondria, strategically located near myofibrils and densely packed with cristae to concentrate ATP generation per unit volume. However, a consequence of dense inner membrane (IM) packing is that restricted metabolite diffusion inside mitochondria may limit ATP production. Under physiological conditions, the flux of ATP synthase is set by ADP levels in the matrix, which in turn depends on diffusion-dependent concentration of ADP inside cristae. Computer simulations show how ADP diffusion and consequently rates of ATP synthesis are modulated by IM topology, in particular (i) number, size, and positioning of crista junctions that connect cristae to the IM boundary region, and (ii) branching of cristae. Predictions are compared with the actual IM topology of a cardiomyocyte mitochondrion in which cristae vary systematically in length and morphology. The analysis indicates that this IM topology decreases but does not eliminate the “diffusion penalty” on ATP output. It is proposed that IM topology normally attenuates mitochondrial ATP output under conditions of low workload and can be regulated by the cell to better match ATP supply to demand.

## 1. Introduction

The process of mitochondrial energy transduction takes place on a highly convoluted inner mitochondrial membrane (IM) nested inside a smoother outer membrane (OM). The transfer of electrons from substrates to oxygen within respiratory complexes on the IM is coupled to transport of protons across the membrane from the internal matrix space to outer compartments, establishing a chemiosmotic potential or proton motive force (pmf) [1]. Since the respiratory complexes are located mainly on invaginated regions of the IM called cristae [2], most of the protons are pumped into intracristal spaces (ICS). The pmf is composed of a chemical potential established by a pH differential (alkaline inside) and a four-fold larger electrical potential (ψ_m_, negative inside). ψ_m_ directly drives several mission-critical processes of mitochondria, including transport of adenine nucleotides across the impermeable IM and powering of the F_1_F_0_–ATP synthase that phosphorylates ADP to make ATP, the cell’s energy currency. This nanomachine is located primarily on curved regions of cristae, with ADP phosphorylation occurring on the F_1_ domain that protrudes into the matrix.

The cristae on which the electron transfer and ATP synthase complexes reside are not random folds but invaginations, formed at membrane necks (loci of reversed membrane curvature) in the boundary (peripheral) region of the IM adjacent to the OM (called the IBM). As a consequence, reactions catalyzed by these complexes can be expected to be highly compartmentalized and dependent on solute diffusion. For example, there is experimental evidence that concentration gradients of protons (which can regulate the rate of ATP synthesis) occur inside cristae as well as between the intracristal space (ICS) and the gap between the IBM and the OM [3], cf. [4]. Likewise, there is experimental evidence that remodeling of the IM involving fusion of crista compartments and widening of crista necks increases the mobility of a soluble protein (cytochrome *c*) between the ICS and the OM/IM gap [5].

The latter IM topological change was described in isolated mouse liver mitochondria treated with pro-apoptotic tBid using transmission electron microscopic tomography (ET), a three-dimensional (3-D) nano-scale imaging technique first applied to mitochondria 30 years ago [6,7,8]. The widespread use of ET, including application to frozen-hydrated cells and tissues (e.g., [9,10,11,12,13,14]), and newer 3-D electron-imaging techniques, such as serial block face scanning electron microscopy (e.g., [15,16]), along with exciting advances in super-resolution light microscopy (reviewed in [17]), particularly of live cells (e.g., [18,19]), has caused a resurgence in interest about how IM topology might be “regulated by the cell to optimize mitochondrial performance in response to different stimuli” [20] (see [21,22,23,24,25]). Key to progress in this area has been the rapidly expanding knowledge of the proteins involved in forming and regulating the inner-membrane necks, first named pediculi cristae [26] and later crista junctions [7], CJ, as well as in establishing the long-range folding of the IM. Mitochondrial membrane-shaping proteins include subunits of MICOS, the “mitochondrial contact site and cristae organizing system” involved in forming the CJ necks [27,28,29,30,31,32]; Opa1/Mgm1, an inner-membrane GTPase involved in mitochondrial fusion that also appears to act as a CJ gate [11,14,33,34]; and the ATP synthase itself, which can dimerize and align in linear or spiral rows that may stabilize or possibly induce the IM to fold into lamellar or tubular morphologies [10,35,36,37].

It is clear that, since the initial endosymbiotic event at the origin of the eukaryotic lineage, mitochondria have substantially integrated into many cellular metabolic and signaling pathways. In the process, mitochondria have evolved into a major signal processing hub of the cell, with direct involvement in a host of physiological processes, e.g., [38,39]. Likewise, impairment or dysregulation of mitochondrial function is directly linked to a spectrum of rare neurological and neuromuscular syndromes [40] and implicated in more common chronic diseases, including but not limited to neurodegenerative diseases, cancer, and heart failure [41]. Understanding the factors that contribute to optimum mitochondrial function is, therefore, a topic of considerable clinical value.

While mitochondria have many physiological roles, there is little doubt that, inside the cells of tissues like muscle, brain, and kidney, their main role is energetic, namely production of ATP. This is supported by observations that packing density of the inner membrane, the scaffold for the chemiosmotic machinery, generally varies in a manner that correlates with the energy demands of the cells and tissues in which they reside (reviewed in [42]). In principle, denser IM packing should provide more ATP production per unit volume occupied by mitochondria, as exemplified by the extreme IM packing inside the mitochondria of insect flight muscle and metazoan heart muscle (e.g., [43,44]). However, gains in ATP production expected from extreme crista packing could be offset by restricted diffusion of metabolites between internal compartments [6,8,45]. This “crista conundrum” was addressed in our previous study utilizing computer simulations of ATP output by model mitochondria that vary in crista size and morphology [46]. The results predict that, as the extent of IM folding increases, restricted internal diffusion can cause ADP to be depleted inside cristae during physiological steady states. This depletion results in reduced rates of ADP transport into the matrix via the adenine nucleotide translocase on the IM, leading to lower matrix ADP levels and corresponding decline in flux of the ATP synthase. Under the conditions of the simulations (chosen to mimic physiological conditions), restricted diffusion results in large “energy penalties” (reduction in ATP output) of up to 25% for the models, and even greater when results are extrapolated to the dimensions and extent of IM folding of cardiomyocyte mitochondria.

The cardiomyocyte is an example of a cell that must maximize ATP generation to function while maintaining a large reserve to meet extreme demands. The prevailing view is that the ATP pool is relatively constant under normal workloads but can be exhausted within a few seconds (after a few heart beats) if not continuously replenished [47,48]. These cells display extreme reliance on mitochondrial ATP production (95% vs. 5% from less efficient glycolysis), fueled predominantly by oxidation of fatty acids [49], which yield three times more ATP per fully oxidized molecule than glucose. Therefore, it seems likely that IM topology in cardiomyocytes has evolved to reduce barriers to internal diffusion that would otherwise limit rates of ATP production. In a recent electron tomographic analysis of mitochondria inside rat cardiomyocytes, we found that mitochondria in proximity to myofibrils (where local energy demand is greatest) predominantly contain lamellar rather than tubular cristae [50]. While simulations indicate that tubular morphology has lower “energy penalties” than lamellar for small cristae, this advantage reverses with increasing crista volumes such as found in muscle mitochondria [46,50].

In the current paper we use computer simulations to explore how additional aspects of inner membrane topology may affect rates of mitochondrial ATP synthesis, in particular, the size, number, and location of CJ necks and extent of crista branching. The model predictions are then compared with the 3-D structure of a unique mitochondrion whose pronounced IM polarity facilitates analysis of variations in IM topology as a function of crista size.

## 2. Materials and Methods

### 2.1. “Virtual Mitochondrion” Simulations

Computer simulations of mitochondrial ATP synthesis inside two- and three-dimensional (2-D and 3-D) spatial models for the inner mitochondrial membrane were run on the Virtual Cell platform [51,52] using the “reduced” metabolic model described in [46]. The model is derived from a more complete mathematical description of energy metabolism [53], largely by setting the chemiosmotic driving force and several other parameters (such as phosphate concentration) as constants. As described in [46], steady-state intracristal [ADP] distributions and reaction fluxes obtained with the “reduced” metabolic model are consistent with those of the complete model, which in turn are consistent with experimental results for mitochondrial oxidative phosphorylation. Thus, the “reduced” model was deemed useful for rapidly screening the effects of varying crista geometries on mitochondrial ATP generation. The “reduced” model involves the following equations for fluxes of adenine nucleotide translocase and F_1_F_0_-ATP synthase [53,54]:

(1) $$J(ANT)=V_{ANT}\frac{1-\frac{[ATP^{4-}{]}_{e}[ADP^{3-}{]}_{m}}{[ADP^{3-}{]}_{e}[ATP^{4-}{]}_{m}}\exp\left(\frac{-F\psi_{m}}{RT}\right)}{\left\{1+\frac{[ATP^{4-}{]}_{e}}{[ADP^{3-}{]}_{e}}\exp\left(\frac{-f_{P}F\psi_{m}}{RT}\right)\right\}\left\{1+\frac{[ADP^{3-}{]}_{m}}{[ADP^{4-}{]}_{m}}\right\}}$$

where V_ANT_ is the maximum velocity of the translocase, F is the Faraday constant, $\psi_{m}$ is the transmembrane electrical potential, $f_{P}$ is the fraction of effective membrane potential for the translocase (set to 0.5 [54]), and the ionic forms of ADP and ATP are fractions of the total ATP and ADP concentrations (inside the matrix, m, and external to the matrix, e); and

(2) $$J(AS)=V_{AS}\frac{\frac{{\left[ADP\right]}_{m}}{K_{ADP}}\frac{[P_{i}{]}_{m}}{K_{Pi}}-\frac{{\left[ATP\right]}_{m}}{K_{ATP}}}{\left(1+\frac{{\left[ADP\right]}_{m}}{K_{ADP}}\right)\left(1+\frac{[P_{i}{]}_{m}}{K_{Pi}}\right)+\left(\frac{{\left[ATP\right]}_{m}}{K_{ATP}}\right)}\frac{[\psi_{m}{]}^{8}}{K_{V,ATP}^{8}+[\psi_{m}{]}^{8}}\left[1-e^{\frac{-[Ca^{2+}{]}_{m}}{K_{Ca,ATP}}}\right]$$

where V_AS_ is the maximum velocity of the synthase, K_ADP_ is the binding constant for ADP, K_ATP_ is the binding constant for ATP, K_Pi_ is the binding constant for P_i_, K_Ca,ATP_ is the Ca^2+^ binding constant, and K_V,ATP_ is the membrane potential yielding half-maximal ATP production (all parameters set as defined in [53]). In addition, a “surrogate kinase” reaction was included that represents cycling of ATP ↔ ADP outside the matrix via such enzymes as adenylate and creatine kinases. The rate of this reaction is represented by the mass action equation

(3) $$V_{SK}=k_{f}{\left[ATP\right]}_{e}-k_{r}{\left[ADP\right]}_{e}[{P_{i}]}_{e}$$

This reaction was tuned in simulations to be near equilibrium (sensitive to local ADP level) by setting the forward rate constant, k**_f_**, to 0.009 ms^−1^ and the reverse rate constant, k**_r_**, to 1 mM ms^−1^. In simulations, starting matrix [ATP] and [ADP] were typical resting state values (0.44 and 0.72 mM), as was the cytosolic [ATP] value (4.0 mM) [53]. Cytosolic [ADP] spanned a range from low to moderate workload in mammalian cardiomyocytes (0.0185–0.074 mM [55,56]) and, along with cytosolic [ATP], was held constant at the boundaries. The IM electrical potential, ψ_m_, was set to 172 mV and all other parameters and conditions were set as in [46], including uniform distribution of enzyme and transporter activities on the inner membrane surface and inside cristae (discussed in Section 4 and in [46]). Under these conditions, maximum values for J(AS) obtained with the “reduced” model (~100 molecules ATP/ms/μm^2^) are similar to those determined experimentally. For example, the maximum rate determined for isolated cardiomyocyte mitochondria in [57], 1.3 mM ATP/s per liter of cells, is equivalent to ~70 molecules ATP/ms/μm^2^, based on mitochondria occupying around 30% of the cardiomyocyte volume and an inner membrane surface-to-volume ratio of 37 μm^2^/μm^3^ [44].

### 2.2. Three-Dimensional Analysis of the Mitochondrial Inner Membrane

#### 2.2.1. Electron Tomographic Reconstruction

The electron tomogram used in this study was from a dataset produced by the Advanced Electron Microscopy Group of the Wadsworth Center, New York State Department of Health, in Albany, NY, USA. Complete details about specimen preparation and data collection are provided in [50]. Briefly, the specimen was a ~200 nm thick section of a rat cardiomyocyte that had been fixed, stained and plastic-embedded according to established protocols [58]. The tomographic reconstruction was computed by weighted back-projection [59] of a tilt-series dataset (+60° to −60° at 1° increment) recorded at 400 kV acceleration voltage and zero energy loss. The reconstructed volume has dimensions 1024 × 1024 × 98 voxels, each (1.8 nm)^3^, in x–y–z where z is the direction of the electron beam normal to the untilted section plane (x–y), and y is the direction of the tilt axis. The tomogram contains a 176 nm-thick segment through a mitochondrion with a roughly circular profile in the x–y plane that decreases gradually in diameter along the z axis from approximately 1.4 μm to 1.3 μm. The tomogram can be viewed as a ‘z–stack’ of 98 x–y slices in the video loop provided in Supplementary Materials. In what follows, a subvolume of 57 x–y slices from the middle of the reconstruction was used, where overall resolution and contrast is greatest. This represents a central slab with thickness of ~130 nm, based on ~20% shrinkage of plastic sections in the electron beam [60].

#### 2.2.2. Segmentation and Surface Rendering of the Mitochondrion

Resolution and contrast in electron microscopic tomograms are affected by factors related to the specimen and electron optics (e.g., heavy metal “stain grain”, locally uneven staining, scattering from gold fiducial markers, large inelastic scattering background), as well as by directional resolution loss due to the “missing wedge” in tilt series data collection [59]. To interpret these 3-D images, preprocessing is used to reduce noise and enhance features of interest, in this case, mainly curved crista membrane surfaces. A spatial filtering procedure was developed and implemented in Microscopy Image Browser (MIB, version 2.83; [61]) that expedited resolving the membranes in the reconstruction. The protocol involves serial application of Gaussian and Hessian algorithms: anisotropic diffusion [62], Frangi [63], and median [64] filters. After spatial filtering of x–y slices, density thresholding was applied to regions of interest (ROI) comprising local clusters of cristae. The output profiles (threshholded slices) of each ROI were used to generate membrane segments that were interpolated in the z-direction and 3-D rendered using the “isosurface” function (based on the “walking cube” algorithm) in MATLAB (version 9.12 R 2022a, Mathworks, Inc., Natick, MA, USA). The file was then ported to Blender (version 2.93.1, https://www.blender.org/, accessed on 12 December 2024) for final 3-D mesh build and clean-up using BlendGAMer (version 2.0.7). Three criteria were used: watertight (enclosed surface integrity), non-manifold (no overlapping vertices and polygons), and triangulated (optimally aligned) [65]. Crista junction “necks” generally aligned with corresponding openings in the IBM but the surfaces were not combined to form one object, which would have caused loss of color-coding. In most models cristae were sealed at the upper and lower faces of the subvolume in order to more easily discern which pairs of closely apposed membrane surfaces form individual crista compartments.

Note that numbering of cristae in this report differs from that in the earlier publication [50], based on re-analysis of connectivity: crista C6 was not previously designated as branched and three closely packed crista clusters previously scored as single interconnected cristae are now designated as seven individual cristae (C10–16). Membrane crowding and resolution limitations complicated determination of membrane connectivity in some regions of the tomogram, necessitating manual curation.

## 3. Results

### 3.1. Computational Modeling of the Modulation of ATP Synthase by Inner Membrane Topology

#### 3.1.1. Effects of the Number and Positioning of Crista Junctions on ATP Production

**Figure 1:**
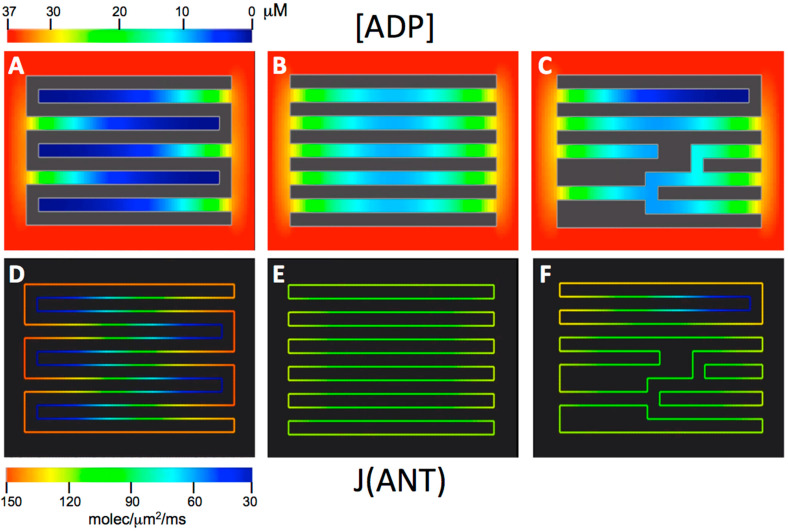
ANT flux inside cristae mirrors ADP depletion. Results of computer simulations of mitochondrial ATP synthesis for inner membrane spatial models with uniform unbranched cristae connected to the inner boundary membrane by crista junctions at one end (**A**,**D**) or both ends (**B**,**E**); and for spatial models with cristae that vary in branching and crista junction distribution (**C**,**F**). Upper frames are maps of ADP concentrations inside cristae and in the cytosol. Lower frames are maps of the flux of the Adenine Nucleotide Translocator (ANT) on the inner membrane surface. Cytosolic [ADP] is set to 0.037 mM on the outer boundary. Cristae are 20 nm wide, spaced 20 nm apart in the uniform models. Vertical and horizontal directions are rendered at different scales in the maps: vertical and horizontal boundaries are 0.30 and 0.76 μm long, respectively.

In our previous computational study of the influence of crista morphology on the flux of ATP synthase [46], cristae were connected to the IBM at single necks or crista junctions (CJ). While this is often the case in mitochondria of liver and other tissues (e.g., [5,6,45]), multiple CJ openings per crista are also common, e.g., in muscle and neuronal mitochondria [7,50,66]. For the current investigation, 2-D spatial models employed in the previous study (Figure 1A,D) were modified by addition of a second CJ to the opposite (trans) end of each crista (Figure 1B,E) [46]. In addition to these uniform topology spatial models, a variable topology model was used containing cristae with different lengths and topologies (connectivity and branching), shown in Figure 1C,F.

**Figure 2:**
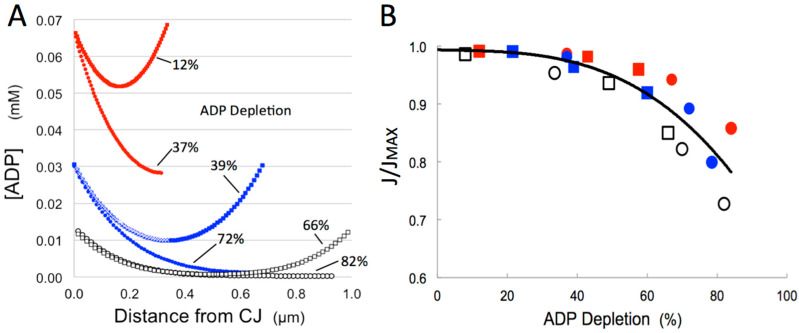
Impact of intracristal ADP depletion on flux of ATP synthase. Results of computer simulations of mitochondrial ATP synthesis for inner membrane spatial models with uniform unbranched cristae as in Figure 1A,B. Symbols in the curves correspond to cristae connected to the inner boundary membrane by crista junctions at one end (circles) or both ends (squares), color-coded by cytosolic ADP levels of 0.074 mM (red solid), 0.037 mM (blue solid), and 0.0185 mM (black open symbols). (**A**) Representative plots of intracristal ADP gradients as a function of distance from CJ openings for varying crista lengths (0.32, 0.64, 0.96 μm, as indicated by the extent of the curves). Intracristal ADP depletion is indicated for each condition, calculated in terms of the areas under each curve. (**B**) Plot of the variation in flux of ATP synthase, J, measured relative to the flux in models absent cristae, J_MAX_, for the entire data set (18 conditions). Crista lengths increase from left to right for each set of three identical symbols.

Typical maps of intracristal ADP concentration, [ADP]_ICS_, during steady-state ATP production are presented in Figure 1A–C for the three models, with cytosolic ADP concentration, [ADP]^O^_CYT_, set at 0.037 mM and maximum crista length, L_CRIS_, of 0.6 μm. Profiles of [ADP]_ICS_ along the cristae for these and other conditions are plotted in Figure 2A. The standing ADP gradients are established inside cristae at steady state by three processes: diffusion from the cytosolic pool of ADP through the CJ “bottleneck”, local transport of ADP into the matrix via ANT, and local hydrolysis of ATP by intracristal kinase activity (see Figure 2 in [46]). The extent of ADP depletion (measured as areas under the curves, labeled in Figure 2A) clearly increases with crista length and is greater for cristae with only one CJ opening to the cytosolic ADP pool. Empirically, the concentration of ADP inside cristae with CJ openings at both ends is fit (R^2^ ≥ 0.99) by the parabola-like quadratic equation:

(4) $$\left[ADP\left(x\right)\right]=[ADP\left(0\right)]-ax(L_{CRIS}-x)$$

where x is the distance from the CJ opening at x = 0, L_CRIS_ is crista length, and

(5) $$a=k\left[ADP\left(0\right)\right]/L_{CRIS}$$

where k varies systematically in a range (1–3.7 μm^−1^) that defines the steepness of the ADP gradient. As illustrated by the white curve in Figure 2A, ADP gradients inside cristae with one CJ follow the first half of curves for cristae twice as long with 2 CJs. We can call this the *Rule of 2* for intracristal ADP gradients. In effect, at steady state, one long crista with openings at both ends is equivalent to two cristae half as long, back-to-back.

**Figure 3:**
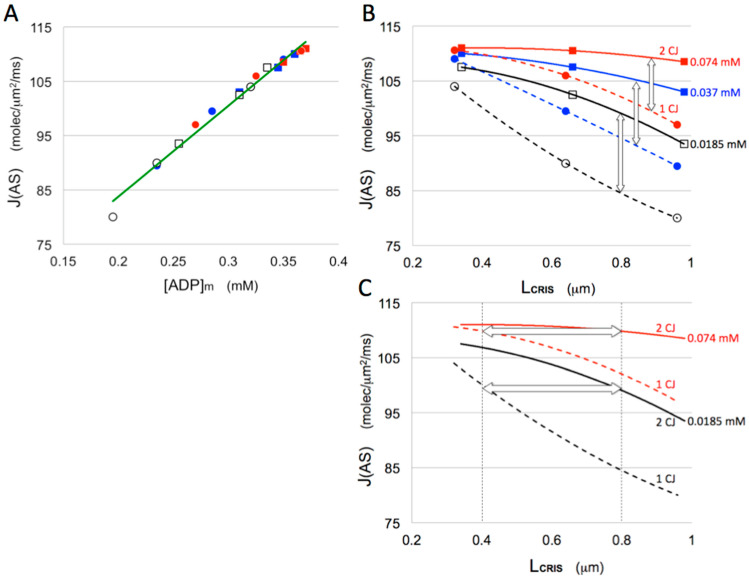
Dependence of flux of ATP synthase on matrix [ADP] and crista length. Results of computer simulations of mitochondrial ATP synthesis as in Figure 1 and Figure 2. Symbols and colors have the same meaning as in Figure 2. (**A**) Plot of flux of ATP synthase, J(AS), as a function of matrix ADP concentration, [ADP]_m_. Dashed green line is a linear fit to the data. (**B**,**C**) Plots of J(AS) as a function of crista length, L_CRIS_, with dashed curves corresponding to cristae with a single crista junction (1 CJ) and solid curves to cristae with crista junctions at both ends (2 CJ). Vertical white arrows in B indicate the shift in the flux caused by addition of a second *trans* CJ to cristae. Horizontal white arrows in C show that the flux for cristae with 2 CJs is approximately equal to the flux for a crista half as long (0.4 vs. 0.8 μm) with only 1 CJ (the *Rule of 2*) across the range of cytosolic [ADP] levels used in the simulations.

Under the conditions of the simulations, the standing gradients of [ADP]_ICS_ are mirrored by gradients in the flux of ANT, J(ANT), along the inner membrane as evidenced in the 2-D maps of Figure 1D–F. The net effect is reduction in steady-state matrix ADP levels, [ADP]_MAT_, which in turn down-regulates ATP synthesis. These effects are represented graphically in Figure 3. There is a smooth, nearly linear, dependence of the flux of ATP synthase, J(AS), on [ADP]_MAT_ over the range in the simulations (0.18–0.37 mM) (Figure 3A). Plots of J(AS) vs. L_CRIS_ in Figure 3B illustrate the effect of increasing intracristal diffusion distances on ATP production. In mitochondrial models with single CJ openings per crista, fluxes of ATP synthase drop significantly, by 13–23%, as crista lengths increase from 0.3 to 0.9 μm. However, the addition of a second CJ to the opposite (*trans*) end of each crista reduces this “diffusion penalty” on rates of ATP synthesis to 3–13% (see white arrows in Figure 3B). Also, due to the close relationship between intracristal [ADP] and flux of ANT (Figure 1), which sets the ADP level in the matrix, the *Rule of 2* for ADP gradients inside cristae has a useful corollary, indicated by the white arrows in Figure 3C: the flux of ATP synthase supported by cristae with two *trans* CJs is approximately equal to that for cristae half as long with a single CJ. Clearly, there is a major bioenergetic advantage to adding a *trans* CJ to cristae as mitochondrial dimensions increase.

#### 3.1.2. Increase in Width of Crista Junctions Increases Rate of ATP Synthesis

To determine how the size of crista junction openings influences ATP synthesis requires the use of 3-D spatial models. In the previous study simulations were run using crista models with lamellar morphology (parallel flat walls 150 nm wide, with variable lengths, spaced 20 nm apart) connected to the IBM with single narrow (20 nm wide) CJ [46]). For this study we included lamellar cristae with no CJs and single slit-shaped CJs equal in width to the cristae. The first new topology (detached cristae) represents the extreme situation of no diffusion of ADP into cristae from the cytosol, while the second topology (baffle cristae) represents the opposite extreme, minimizing the CJ diffusional bottleneck. All three topologies—narrow tubular CJs, wide slit-like CJs, and detached cristae—are commonly observed in tomograms of actual mitochondria. In general, the relationships among intracristal ADP gradients, matrix ADP levels, and ANT fluxes in simulations run with these and related 3-D models [46] are similar to those obtained with the 2-D models which, mathematically, correspond to cross sections through 3-D models with baffle cristae.

The 3-D models used for the simulations have ratios of crista membrane surface area (S_CRIS_) to IBM surface (S_IBM_) in the range 1.0–1.8 [46], which is on the low end of the range observed in tissues like liver, brown fat, and retinal rods (S_CRIS_/S_IBM_ = 2.3, 4.9 and 5.2, respectively [67]) and reach 10 or higher in cardiac muscle [44,50]. It is a relatively simple matter to isolate the topology-dependent component of ATP synthesis from the total flux in the models as follows. At steady state,

(6) $${J(AS)}_{MODEL}\sim {J\left(ANT\right)}_{MODEL}=J_{MAX}-\Delta J_{DIFF}$$

where J_MAX_ is the flux in the absence of cristae, easily determined with a fourth spatial model lacking cristae entirely, and ΔJ_DIFF_ is the “diffusion penalty” (called the “energy penalty” in [46]) for a given IM topology, i.e., reduction in flux due to restricted internal ADP diffusion. The steady-state fluxes in the models can be simply defined in terms of contributions from the IBM and cristae using the relative ratios of IBM and crista membrane surface areas (S_IBM_, S_CRIS_) to total IM surface area (S_IM_):

(7) $$J_{MODEL}=(S_{IBM}/S_{IM})J_{MAX}+(S_{CRIS}/S_{IM}{)J}_{CRIS}$$

thus, the topology-dependent contribution of cristae to ATP production is

(8) $$J_{CRIS}=(S_{IM}/S_{CRIS})J_{MODEL}-(S_{IBM}/S_{CRIS}{)J}_{MAX}$$

**Figure 4:**
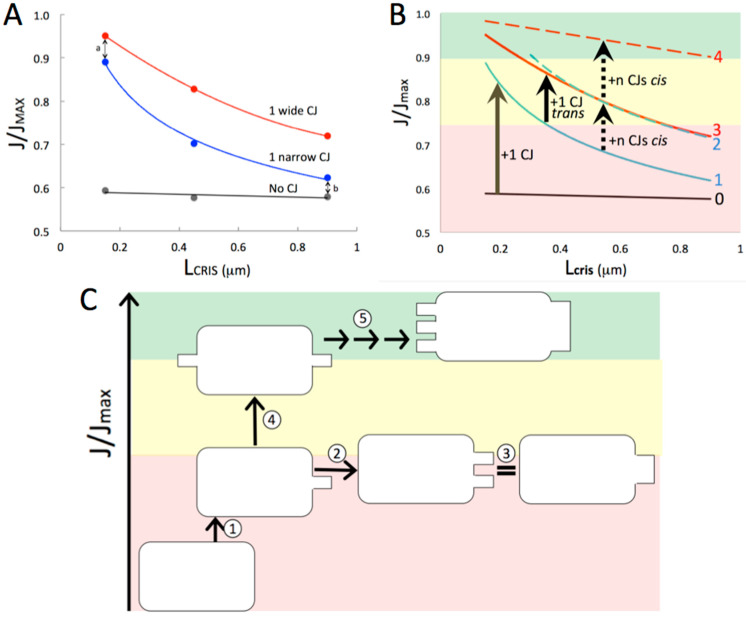
Dependence of flux of ATP synthase on crista junction number, distribution, and size. (**A**) Results of computer simulations of ATP synthesis employing 3-D spatial models for lamellar cristae. Curves correspond to the relative flux of ATP synthase as a function of crista length, L_CRIS_, for cristae with no junctions (No CJ), one CJ with a width of 20 nm (1 narrow CJ), and one CJ with a width of 150 nm (1 wide CJ), for L_CRIS_ = 0.15, 0.45 and 0.9 μm. Arrows labeled “a” and “b” indicate the gaps between the indicated curves at small and large L_CRIS_. (**B**) Like A but dashed curves added for addition of a *trans* CJ to cristae with one CJ, calculated using the *Rule of 2*. Solid vertical arrows indicate the shifts in relative flux of ATP, J/J_MAX_, associated with adding the second CJ. Broken vertical arrows indicate the incremental gains in J/J_MAX_ corresponding to sequential addition of narrow CJs to either side of the cristae, which is equivalent to widening the cristae junctions. Color overlays indicate zones of fast (green), moderate (yellow), and slow (red) ATP synthesis, as described in the text. (**C**) Cartoon illustrating how J/J_MAX_ increases with successive changes in topology for a crista of constant L_CRIS_, from (1) addition of a single CJ to a detached crista, (2) adding a second CJ to the same side, which is equivalent to (3) widening the CJ, (4) addition of a CJ to the opposite (*trans*) side of the crista, and (5) widening and adding more CJs to both ends of the crista.

Predicted variations in J(AS)_CRIS_ under physiological conditions in resting cardiac muscle as a function of crista length (150–900 nm) are plotted in Figure 4A for the three topological models: detached cristae, single narrow CJ, and single wide CJ. The strong influence of internal diffusion on ATP synthesis is apparent in the wide variation in fluxes (almost 40%) across the range of crista lengths and topologies. Interestingly, the decrease in flux of ATP synthase (“diffusion penalty”) for short cristae with one narrow CJ is close to that for cristae with a single wide slit CJ opening (arrow “a”), but increases with crista length to just above the flux for detached (no CJ) cristae (arrow “b”). From a bioenergetic perspective, as cristae get larger, having one narrow CJ is about the same as having none, a suboptimal situation in a tissue like cardiac muscle.

Figure 4B is a schematic summarizing the predictions from the computer simulations, and Figure 4C is a cartoon illustrating the corresponding changes in crista topology. Fluxes of ATP synthase (relative to that in the absence of diffusion restrictions, J_MAX_) can be considered to fall into three “speed zones”: fast (green) for flux ≥ 0.9 × J_MAX_, moderate (yellow) for flux = (0.9 − 0.75) × J_MAX_, and slow (red) for flux < 0.75 × J_MAX_. Detached cristae operate at about 0.6 × Jmax (solid black line). Connecting a crista to the IBM at a single narrow CJ (solid blue curve) improves matters a bit: fluxes move from red into the yellow zone for short cristae, but drop back into the red zone as lengths approach 0.4 μm. Increasing the width of the CJ opening from 20 nm to 150 nm (the width of the crista) improves matters considerably (solid red curve), keeping fluxes in the yellow zone until lengths reach ~0.8 μm. Note that widening one CJ opening is equivalent to adding multiple (n) narrow (20 nm wide) CJs to the same (*cis*) side of the cristae, where n = 1 to 6. It turns out that the gain in flux from maximizing diffusion at one end of a crista is equivalent to adding a second narrow CJ at the *trans* side (dashed blue curve). In order to keep the rate of ATP synthesis in or near the “fast” zone across the full range of crista lengths, diffusion bottlenecks have to be minimized at both ends of cristae (dashed red curve).

#### 3.1.3. Effects of Crista Branching on the Rate of ATP Synthesis

The “variable topology” spatial model in Figure 1 includes a large crista with a CJ at both ends, plus two side branches with lengths (L_B_) of 1/3 and 1/4 the length of the main crista (L_CRIS_), each connected to the IBM. The steady-state intracristal ADP and J(ANT) gradients in these side branches closely match those in the central crista “trunk” (Figure 1C,F), following equation (4). Since the maximum diffusion paths in branches are shorter than in the “trunk” (L_B_ < L_CRIS_/2), the extent of ADP depletion and corresponding “diffusion penalties” are smaller than those of the full-length cristae. For example, ADP depletion inside a crista branch with L_B_ = L_CRIS_/4, off a “trunk” with L_CRIS_ = 0.9 μm and cytosolic ADP set at 0.0185 mM is 35%, compared with 66% for the full-length crista “trunk” (upper white curve in Figure 2A). This is a significant difference since J(AS) drops steeply when intracristal ADP depletion reaches 60% or more (Figure 2B). In effect, adding branches to cristae has the effect of shortening the “average” crista length inside a mitochondrion. However, branching will meaningfully impact flux of ATP synthase only when L_CRIS_ is large, branch lengths are short (L_B_ < L_CRIS_/2), and the summed surface area of branches approaches that of the main crista “trunk”. No reduction in “diffusion penalty” accrues from Y- or X-shaped branch points at the middle of cristae, although such branching may optimize another important parameter, namely crista packing (see Section 3.3). With these provisos, large-scale branching of cristae is a topological strategy that can contribute to maximizing mitochondrial ATP output.

### 3.2. Topology of the Inner Membrane in a Cardiomyocyte Mitochondrion

In a prior report, a database of 3-D mitochondrial structures was described, generated by electron tomographic reconstruction of plastic sections of rat cardiomyocytes [50]. Mitochondria were not selected randomly but meant to represent the diversity of crista morphologies in the cells. Thus, while the number of tomograms in any given morphologic class did not represent its frequency of occurrence, correlations of crista morphology (lamellar or tubular) with crista packing density and subcellular location of the mitochondria could be meaningful and, in fact, were found to be statistically significant. In particular, mitochondria with lamellar cristae had the densest crista packing and were dominant in regions adjacent to myofibrils, where local energy demand is expected to be greatest [68,69,70]. The dataset also contained mitochondria with crista subclasses (“transitional” and “swollen”) that suggested a possible remodeling pathway between lamellar and tubular morphologies. The cristae in one “transitional” mitochondrion exhibited marked polarity in size and morphology (from simple lamellar to branched lamellar to tubular) with distance from a specific point on the IBM. This unique mitochondrion afforded an opportunity to compare theoretical predictions about bioenergetically optimal IM topology (Section 3.1) with reality. In particular, are there systematic changes in IM topology as a function of crista length consistent with reducing internal limitations to diffusion?

**Figure 5:**
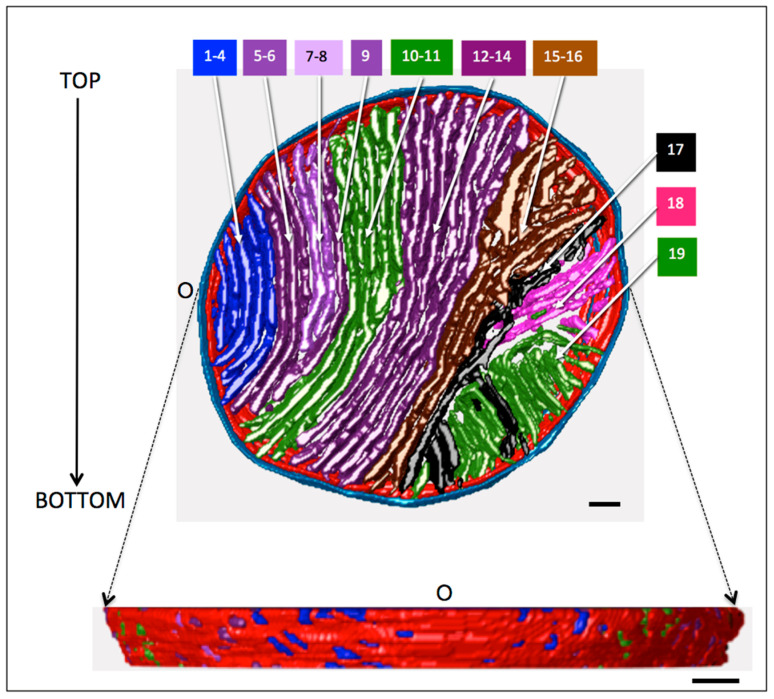
Model of the membrane surfaces in a large cardiomyocyte mitochondrion, obtained by electron tomography. (**Upper panel**): View of the model normal to the tissue section, showing the progression from lamellar to branched lamellar to tubular with distance from a point on the inner boundary membrane (IBM) labeled “O”. In this model the outer membrane (OM) is blue, the IBM is red, and the crista clusters are color-coded and numbered as described in the text. (**Lower panel**): Side view of the model after its rotation in the *x*–*y* plane by ~90° in the counter-clockwise direction and removal of the OM. In this rendering, the crista compartments are sealed for ease of visualization. Scale bars = 100 nm.

An overhead view of the membrane surfaces within the reconstructed region of this mitochondrion (a slab ~130 nm thick) is provided in Figure 5. While there appear to be over 30 crista profiles in this view, 3-D analysis indicates there are 19 unique cristae not interconnected within the section, numbered C1–19 from point “O” on the IBM. The color-coding of cristae in the model reflects regions of interest selected during the image analysis process (see Methods), which was influenced by visual impressions of crista shape and orientation. As noted above, there is a marked polarity in membrane morphology from left to right in Figure 5, with cristae in the left two-thirds of the mitochondrion (C1–16) having predominantly lamellar morphology and those at right (C17–19) displaying more tubular shapes.

#### 3.2.1. Branching of Lamellar Cristae

**Figure 6:**
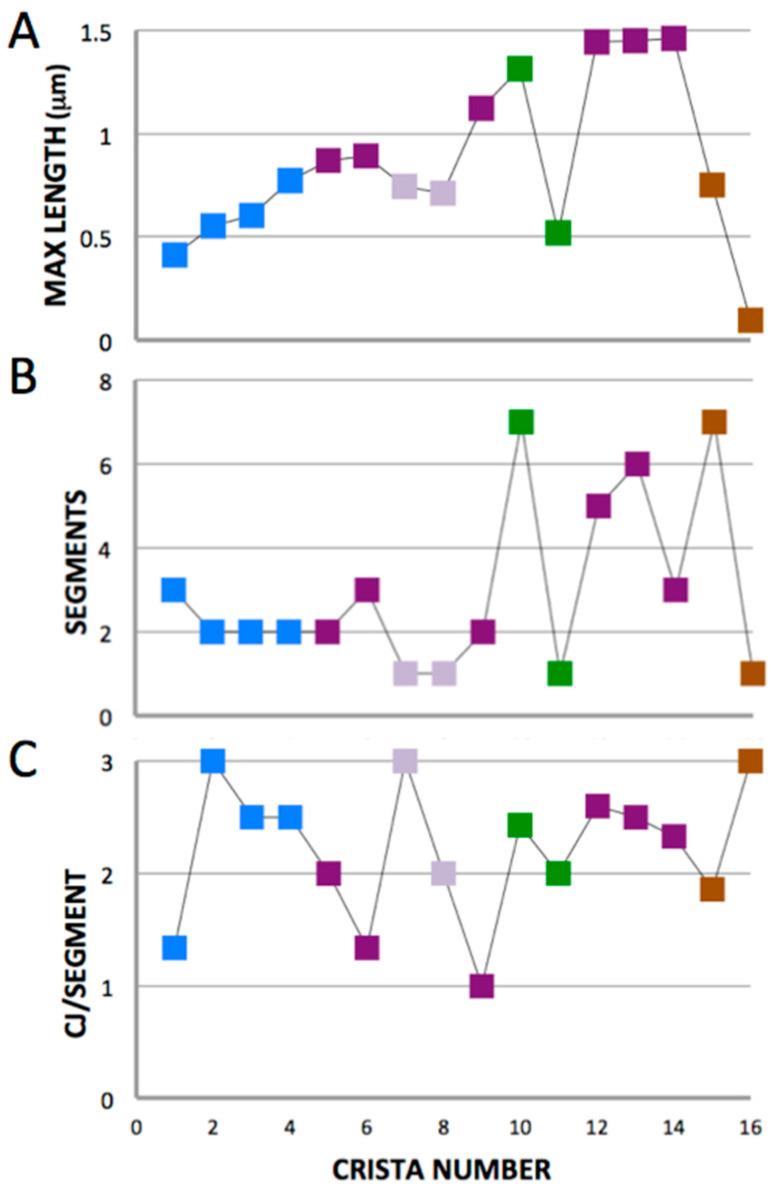
Variation in the length, branching, and junction density of the lamellar cristae. Plots of (**A**) the maximum length of each crista C1–16, defined as the longest membrane surface in the crista; (**B**) the extent of branching of each crista, defined as the number of connected segments that extend to the IBM surface; and (**C**) the average CJ density of each crista, defined as the total number of CJs divided by the number of segments. Symbols for the cristae are color-coded according to the scheme in Figure 5.

**Figure 7:**
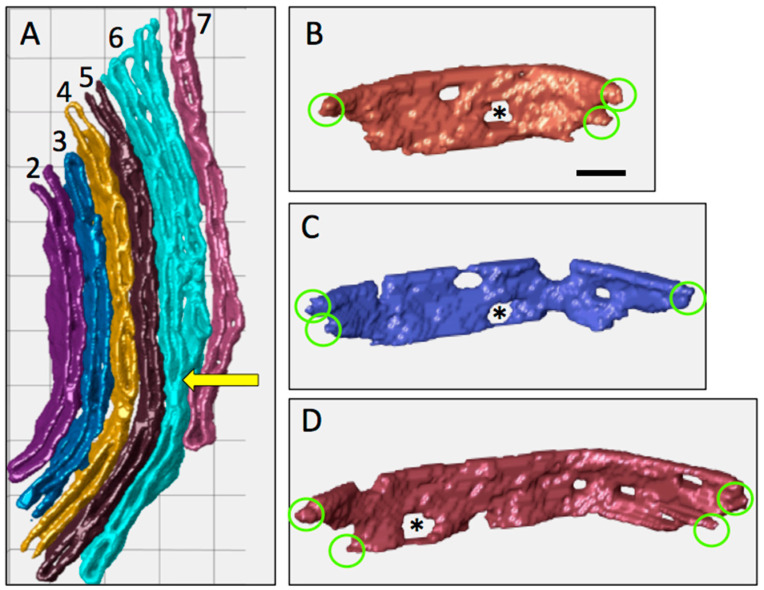
Variations in topology of cristae C2–7. (**A**) In this rendering, the crista compartments are unsealed and IBM and OM are not shown. Color-coding of surfaces does not follow that of Figure 5. The single Y-type branch in C6 is indicated by an arrow. Note the short length of C7, which is one of the few cristae that do not extend to both “top” and “bottom” sides of the IBM. The grid has 50 nm spacings. (**B**–**D**) Side views of cristae C3–5 rendered as sealed compartments as in Figure 5, showing numerous randomly distributed fenestrations (one marked by * in each crista) and typical protruding crista junctions encircled. Surfaces are colored differently than in (**A**). The scale bar in (**B**) is 50 nm.

**Figure 8:**
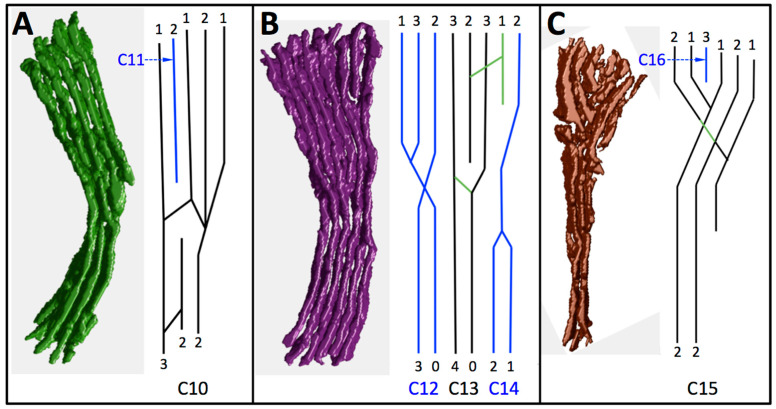
Branching patterns and CJ distribution in cristae C10–16. The branching patterns for each lamellar crista in the clusters (**A**) C10–11, (**B**) C12–14, and (**C**) C15–16 are provided along with the number of CJs on the individual segments. Color-coding of the branching patterns for adjacent cristae alternates between black and blue to avoid confusion. Green lines indicate tubular regions. Lengths of the lines in the branching patterns are only approximate.

With a few exceptions, there is a progressive increase in length of cristae C1–16 (measured as the longest membrane contour in each crista) from 0.41 to 1.46 μm (Figure 6A), with C15 decreasing in length as the cross-section width drops below 1 μm. The zoomed-in view of cristae C2–7 in Figure 7A reveals that C6 is branched, with a single bifurcation to form a Y-shaped compartment. Only one other crista in C1–9 has a similar branch (C1), with the rest unbranched. By contrast, five of the next seven cristae (C10–16) are branched, shown in detail in Figure 8. In four of these cristae (C10, C12, C13, C15), the observed branching is complex, with multiple bifurcations into Y, Ψ, and X patterns at one or both ends. In a few cases lamellar crista segments are interconnected by short transverse tubular segments, as indicated in the branching patterns of Figure 8. Figure 6B is a plot of crista branching, scored as number of segments, for the 16 cristae. (For this purpose, a segment is defined as a crista subregion that extends to the IBM. Thus, an unbranched crista may have one or two segments depending on whether one or both ends span the mitochondrial cross section). Comparison of the data in Figure 6A,B indicates a strong correlation of crista branching with crista length: five of the seven branched cristae are longer than 0.8 μm, and three of the four multi-branched cristae are longer than 1.3 μm.

#### 3.2.2. Number, Positioning and Size of Crista Junctions

The lower part of Figure 5 is a side view of the inner membrane surface that shows numerous “holes”, representing the openings of the crista junctions that connect cristae to the boundary region of the inner membrane (IBM). The openings are generally round with a mean diameter (±standard deviation) of 17 (±5) nm, similar to that reported in mitochondria of other tissues (9–17 nm [67]). In some cases, especially near the “origin”, the openings are elongated, with widths ~17 nm and lengths up to 60 nm. The density of CJs in the IBM surface spanned by lamellar cristae is ~220 per μm^2^, outside the published range for other tissues: 13–174 per μm^2^, corresponding to liver and synaptic neural tissue, respectively [67]. The higher CJ surface density is consistent with the exceptionally dense packing of lamellar cristae in cardiomyocyte mitochondria and suggests extensive connectivity between the IBM and cristae.

Of the 16 lamellar cristae in the mitochondrion, all but four (C7, C8, C11, C16) are long enough to span the mitochondrial cross-section within the reconstructed volume. The four “non-spanning” cristae are unbranched and connected by crista junctions only at the “top” side of the inner boundary membrane (IBM). Of the 12 “spanning” cristae, only one lacks a CJ at both ends, C9, and like the “non-spanning” cristae it is attached only at the “top” side of the IBM. Only one other crista segment is not connected to the IBM, a branch at the “bottom” end of C13 (see branching patterns in Figure 8 which include the number of CJs at the ends of all segments in cristae C10–16). There is no obvious trend in the average number of CJs per segment for cristae C1–16, plotted in Figure 6C. The average number of CJs per segment falls in the range 1.9 to 3 for 13 of the 16 lamellar cristae, with an overall mean of 2.2 (±0.6). Considerably more crista segments extend to the “top” than to the “bottom” side of the IBM (29 vs. 19), but their connectivity to the IBM in terms of CJ/segment at the “top” and “bottom” of the mitochondrial cross section is not significantly different (2.3 ± 0.5 vs. 1.9 ± 1.0, *p* = 0.13 by unpaired *t*-test).

### 3.3. Observed Inner Membrane Topology Reduces “Diffusion Penalties” on ATP Synthesis

Overall, the topology of the inner membrane of this large cardiomyocyte mitochondrion is expected to mitigate but not eliminate the effects of ADP diffusion on ATP production. The single biggest factor is that the majority of cristae (12 of 16) have CJ openings to the IBM at both ends, which has the same effect on intracristal ADP depletion as shortening their lengths (L_CRIS_) by half (Section 3.1.1). If the 16 cristae were unbranched, this would have the effect (from the perspective of ATP production) of reducing the mean L_CRIS_ from 0.86 to 0.53 μm. For mitochondria with uniform cristae of these lengths and single narrow CJ openings (blue curve in Figure 4A), this decrease in “effective” L_CRIS_ would increase the steady-state flux of ATP synthase, J/J_MAX_ (where J_MAX_ is the flux absent diffusion effects), from 0.62 to 0.68, an increase of 10%.

As described in Section 3.1.3, crista branching also can increase the rate of ATP generation by reducing intracristal ADP depletion, but the impact is meaningful only if the branched segments are significantly shorter than half the maximum crista length. In fact, as indicated by the branching patterns in Figure 8, branching usually occurs near the middle of cristae, with only four segments in cristae C10, and C12–15 shorter than 0.5 × L_CRIS_. Thus, the gain in flux (rate/IM surface area) of ATP synthase from crista branching in this mitochondrion is expected to be minimal. However, since branching enables more efficient packing of crista membranes in the center of the circular cross section of this mitochondrion, it increases ATP output (flux × IM surface area) within the mitochondrial volume by 30–40% (based on crista segments added by branching in the region C10–15).

An estimate of the relative steady-state flux of ATP synthase for the mitochondrion of Figure 5 can be calculated as the weighted sum of the contributions from each crista:

(9) $$J/J_{MAX}=\sum_{i=1}^{16}{f_{i}\left[J/J_{MAX}\right]}_{i}$$

where $f_{i}$ is the relative surface area of each lamellar crista (measured in terms of segment lengths, since the crista tend to span the full 130-nm thickness of the section) and ${\left[J/J_{MAX}\right]}_{i}$ is the predicted contribution to the relative flux of ATP synthase by each crista, interpolated from the curves in Figure 4B based on their length and number of CJs on one or both crista ends. The result is J/J_MAX_ = 0.70, compared with 0.62 for the condition that each crista is connected to the IBM at only one end with a single CJ per segment, and 0.58 if all cristae are unattached. The maximum rate obtainable, if all cristae are connected to the IBM at both ends, with CJs having widths equal to the section thickness (i.e., “baffle” cristae), is J/J_MAX_ = 0.93. Thus, the observed IM topology modulates the rate of ATP generation by 60% (from 0.58 to 0.93 × J_MAX_) and the rate set by the observed inner membrane topology (0.70 × J_MAX_) is well below the maximum rate possible under the conditions of the simulations. The value of cytosolic [ADP] used in Figure 4A,B, 0.037 mM, is typically associated with low to moderate workload in mammalian heart muscle [55,56]. If cytosolic ADP levels rise or fall as the result of changes in workload, intracristal ADP depletion will lessen or worsen, respectively (as shown in Figure 2), and the corresponding ATP synthase flux curves will shift up or down, as illustrated in Figure 3B. This raises the possibility that inner membrane topology might serve to attenuate mitochondrial ATP production in large mitochondria when cell workload is low (see Section 4).

Note that this analysis of the modulation of J(AS) by inner membrane topology is based on the dependence of J/J_MAX_ on L_CRIS_ (crista length) in Figure 4, which plots fluxes of ATP synthase supported by uptake of ADP into the matrix via intracristal ANT. This ignores the contribution to J(AS) by ANT activity outside cristae on the IBM, which is unaffected by restrictions to ADP diffusion related to crista size and connectivity to the IBM. Assuming uniform distribution of ANT along the IM surface, the fraction of ANT on the IBM relative to those inside cristae is small for mitochondria with densely packed cristae—only 8% in the case of the cardiomyocyte mitochondrion—since crista membranes account for 92% of the IM surface area. Inclusion of an IBM term in Equation (6) increases J/J_MAX_ from 0.58 to 0.61 for detached cristae, from 0.70 to 0.72 for the observed IM topology, and from 0.93 to 0.94 for maximum crista connectivity to the IBM.

## 4. Discussion

Several inferences may be drawn from this comparison of theoretical effects of membrane topology on ATP production with the actual IM topology of a cardiomyocyte mitochondrion. The simulations predict that IM topology modulates the flux of ATP synthase over a wide range by affecting lateral diffusion of ADP and its uptake into the matrix. The modulation range is considerable—50% or higher for large mitochondria (cristae lengths ≥0.9 μm)—with J(AS) increasing from approximately 0.6 to 0.9 × J_MAX_ (the flux absent diffusion effects) as CJ openings increase in size and number for resting-state heart muscle (illustrated by the cartoon in Figure 4C). The observed IM topology in the mitochondrion analyzed is predicted to reduce, but not minimize, the effects of diffusion on J(AS), increasing the flux by 17% (from approximately 0.6 to 0.7 × J_MAX_) over that with one narrow CJ per crista. Crista branching, unlike the number of CJ openings per crista compartment, systematically increases with crista length, but has little expected impact on J(AS) since only a few branches are significantly shorter than the crista “trunks”. However, crista branching does yield an expected increase of 30–40% in ATP output (= J(AS) × IM surface area) due to more efficient membrane packing within the mitochondrial volume. Combined with the 17% improvement in J(AS) provided by CJ distribution, the predicted topology-dependent increase in ATP output is over 50%.

As with any computer modeling study, the reliability of these conclusions depends on the models, assumptions, and conditions employed. The current study used an established metabolic model [53] implemented on the Virtual Cell platform that was found to generally agree with experimental results (as noted in [46]). Values for cytosolic ADP levels (0.018–0.074 mM) and rate constants for the “surrogate kinase”, which were tuned to be near equilibrium and sensitive to local ADP levels, could be considered to stress effects of restricted internal ADP diffusion on J(AS). However, these conditions are nonetheless “physiological”. The lower cytosolic [ADP] values used (0.018 and 0.037 mM) are within the normal range for mammalian cardiac muscle (0.013–0.055 mM [47]). Similarly, activities of “cycling kinases” (adenylate and creatine kinases) inside mitochondria, as in the cell [71], may be locally attenuated by low levels of metabolites, like AMP and creatine (the latter exacerbated by the enzyme’s relatively low affinity for creatine; K_m_ in the millimolar range [72]), as well as by uneven distribution of the enzymes among micro-compartments. Thus, the modeling conditions used are reasonable and the predicted modulation of J(AS) by IM topology is likely realized in heart muscle cells. Another recent modeling study also has indicated direct impact of IM morphology on mitochondrial ATP output, but from a different set of assumptions [73]. ATP synthase was restricted to tightly curved regions in 3-D spatial models of actual neuronal mitochondria, consistent with the tendency of ATP synthase dimers to form oligomer ribbons on crista tubes and folds [10,37]. The value for cytosolic [ADP] used in these simulations was greater than the maximum value used in the current study (0.1 vs. 0.074 mM) and, combined with the smaller IM dimensions of neuronal vs. muscle mitochondria, would diminish effects of ADP diffusion on J(AS). The results in [73] indicate that neuronal mitochondria models with greater fractions of highly curved IM surfaces have greater steady-state rates of ATP production than models with elongated, flatter cristae. It seems likely the outcome reflects variations in “specific activity” of ATP synthase (activity per total IM surface area) of the models more than effects of ADP diffusion. However, it raises an important question about the actual localization of ATP synthase in cristae that are elongated, wide sheets with relatively small edge regions, as in muscle mitochondria. One possibility is that lamellar IM surfaces have sufficient curvature on the nanoscale to accommodate numerous ATP synthase dimers. We have proposed that ATP synthase dimers may reside on the inner rims of the numerous fenestrations, 20–80 nm wide, that are randomly distributed on the surfaces of lamellar cristae in cardiomyocyte mitochondria, as shown in Figure 7B–D [50] (see also [12,66]). In this case, our modeling assumption of uniform distribution of ATP synthase on the IM surface is appropriate, although effects of non-uniform distribution of ATP synthase and ANT (which controls entry of ADP into the matrix) should be explored in future work.

An important consideration is whether the dependence of mitochondrial ATP output on internal ADP diffusion is relevant to muscle contraction. During the myofibril “cross-bridge cycle”, local cytosolic ADP levels transiently jump when ADP is released from myosin at the end of the “power stroke”. This rise in [ADP]_CYT_ should equal the amount of ATP that subsequently binds to myosin and is hydrolyzed in the next cycle, recently measured at several tenths of a millimolar [74]. Such a spike in [ADP]_CYT_ would increase the J(AS) of nearby mitochondria by reducing the IM topology-dependent “diffusion penalty”, as shown in Figure 2B and Figure 3B, for a 2-fold increase in cytosolic ADP from 0.037 to 0.074 mM. The predicted result is a transient ~30% increase in ATP output during contraction in addition to that resulting from concurrent calcium-stimulation of matrix dehydrogenases. Support for a local increase in [ADP]_CYT_ during muscle contraction is suggested by changes in conformational state of interfibrillar mitochondria during the cardiac mechanical cycle [75]. Although not noted by the authors of the study, mitochondria in the “contracture” phase appear to adopt the “contracted” IM conformation (matrix condensed, cristae expanded) induced by high external ADP levels [76].

Thus, we propose that IM topology is tuned to attenuate mitochondrial ATP production when cellular demand and cytosolic ADP levels are low, such as when time-averaged workload is low or possibly, on a shorter timescale, between myofibril contractions. As cytosolic ADP levels rise, IM topology-related “diffusion penalties” on ATP generation diminish, and might be further reduced to meet demand by IM remodeling. There is growing evidence that cristae are continuously remodeled in all living cells in response to a variety of metabolic and apoptotic stimuli [11,19,24,32,77,78,79,80,81]. Members of the MICOS complex (like Mic26 and Mic13) mediate a wide range of changes in IM topology, including crista fusion and fission that appear to underlie branching (and, as we show, ATP output), with dynamics on the order of seconds [30,32,80,82,83]. Another member of MICOS, MICU1, is a calcium sensor that regulates both activity of the mitochondrial calcium uniporter, MCU (which controls matrix uptake of calcium and subsequent stimulation of ATP production during muscle contraction), and IM topology at crista junctions, with an as-yet undefined relationship between the two functions [80,84]. Opa1/Mgm1, the CJ gatekeeper, regulates the width of CJ openings (which we show directly impacts flux of ATP synthase) as well as mitochondrial fusion [11,14,85], with both functions altered by processing (shortening) of Opa1 by the cellular stress-sensing metalloprotease Oma1 [86]. Thus, the remarkable IM membrane polarity of the mitochondrion in Figure 5 may reflect a lateral gradient of some signal (e.g., ROS, calcium, membrane potential) that varies with local metabolic activity and regulates Opa1, MICU1, and/or other IM topology modulators. Note that the mitochondrion of Figure 5 is in close contact at different depths in the tomogram with other mitochondria with which its cristae are coordinated [66], as well as with sarcoplasmic reticulum at the “top” of the cross-section (see the slice-by-slice video of the tomogram in Supplementary Materials; also Figure 4D in [50]). These adjacencies may have influenced the establishment of an internal (left-to-right) signal gradient responsible for the increase in crista branching (and possibly the remodeling to tubular morphology at far right), as well as the larger number of crista segments extending from upper vs. lower regions of the IBM, due to the tendency of MICOS to localize at ER-OM contacts [87].

From a clinical perspective, if inner membrane topology can “throttle back” baseline ATP production in the healthy heart when energy demand is low, aberrant remodeling of the IM might contribute to energy deficits observed in the failing heart. Continuing advances in large-scale high-resolution in situ 3-D imaging of mitochondria and reliable automated membrane segmentation may make it feasible to screen for systematic perturbations in IM topology in heart failure and other diseases with mitochondrial involvement. Eventually, improved understanding of the factors that shape the inner membrane could lead to new therapies that mitigate the “diffusion penalties” imposed on ATP output by IM topology.

## Data Availability

Electron tomograms used in this study will be made available upon reasonable request. We intend to deposit the data in an appropriate 3D-EM databank in the near future.

## Supplementary Materials

The following supporting information can be downloaded at: https://www.mdpi.com/article/10.3390/cells14040257/s1, Video S1: Slice-by-slice view of tomogram of cardiomyocyte mitochondrion.

## Abbreviations

The following abbreviations are used in this manuscript:

2-D	Two-dimensional
3-D	Three-dimensional
ADP	Adenosine diphosphate
AMP	Adenosine monophosphate
ANT	Adenine nucleotide translocator
AS	ATP synthase
ATP	Adenosine triphosphate
CJ	Crista junction
ER	Endoplasmic reticulum
ET	Electron tomography
IBM	Inner boundary membrane
IM	Inner mitochondrial membrane
MCU	Mitochondrial calcium uniporter
MICOS	Mitochondrial contact site and cristae organizing system
OM	Outer mitochondrial membrane
